# Real-time and digital PCR assays for the detection of *Aspergillus* DNA in contrived and clinical samples—a bi-center evaluation

**DOI:** 10.1128/spectrum.01488-25

**Published:** 2025-10-30

**Authors:** Hanna Kolmeder, Raquel B. Posso, Jan Springer, Hermann Einsele, P. Lewis White, Jürgen Löffler

**Affiliations:** 1 Medizinische Klinik und Poliklinik II, University Hospital Wuerzburg, Wuerzburg, Germany; 2 Public Health Wales Microbiology, and Cardiff University Centre for Trials research, Cardiff, United Kingdom; Universidade do Minho, Braga, Portugal

**Keywords:** bi-center evaluation, *Aspergillus*, digital PCR, real-time PCR

## Abstract

*Aspergillus* real-time PCR (qPCR) is well-established, but limitations persist. These may be overcome by digital PCR (dPCR) technology, with its potential to be more analytically sensitive, more precise, and less affected by inhibition compared to qPCR, all of which could benefit the detection of low DNA concentrations present in many invasive fungal infections. In this study, two well-validated local qPCR assay protocols to detect *Aspergillus* spp. were transformed into a digital format, with analytical and clinical performance assessed by testing contrived (*n* = 30) and clinical samples (*n* = 19) and a range of negative control materials. PCR positivity was determined based on the reported dPCR copy numbers, assay concordance, and correlation between the burdens (copies/μL) measured by both dPCR assays. Both dPCR assays generated 100% agreement across the contrived samples, detecting all the positive samples with a significant positive correlation (rho: 0.9605, *P* < 0.001) between the fungal burdens calculated between assays. When testing clinical samples, the overall qualitative agreement between the dPCR assays was 84%, with discordant and false positive results associated with very low DNA concentrations (one positive partition). There was a strong correlation (rho 0.887, *P* < 0.001) between the DNA concentrations calculated by both dPCR assays when testing clinical samples.

**IMPORTANCE:**

For aspergillosis, available DNA burdens can be low, and quantification is required to differentiate colonization from disease. Real-time PCR (qPCR) has been widely used to aid the diagnosis of aspergillosis, but performance can be hampered when targeting burdens at or near the limit of qPCR detection, and quantification requires the use of standard curves that may be impacted by the sample matrix. The robust cross-platform quantification of low DNA concentration provided by digital PCR (dPCR) platforms offers an enhanced diagnostic option to real-time PCR, with the potential to enhance the molecular diagnosis of this disease. The ability to detect low DNA burdens, coupled with the potential for genotyping, could be further exploited to detect genetic mechanisms of antifungal resistance.

## INTRODUCTION

Invasive aspergillosis (IA) remains a major complication in patients with hematological malignancies and post-allogeneic hematopoietic stem cell transplantation (1, 2), but an expanding number of clinical cohorts are now at risk, including patients suffering from severe respiratory virus infections (3). IA poses a major clinical challenge due to the variable sensitivity and specificity of diagnostic tools, insufficient efficacy of available antifungal therapies, and increasing antifungal drug resistance (2, 4, 5). Therefore, IA is associated with high mortality rates (1, 6) and poor long-term survival (7). Early detection of *Aspergillus* infections has the potential to facilitate a more effective management of invasive disease.

Typically, the classification of IA is based on radiological, clinical, and mycological data (8). With proven IA rarely achieved in clinical practice, most patients attain a probable classification that includes culture, microscopy, galactomannan antigen, and *Aspergillus* DNA detection (8–10). Nevertheless, these tools provide variable sensitivity and specificity depending also on the material tested, which can contain very low concentrations of the target (11, 12).

While *Aspergillus* real-time PCR (qPCR) is well-established, limitations persist, some of which are addressed by digital-PCR (dPCR) technology, where the PCR reaction is partitioned in either droplet (ddPCR) assays or non-droplet PCR assays, such as chip or nanoplate digital PCR. Digital-PCR has the potential to be more sensitive and more precise than qPCR, especially for the detection of low DNA amounts. Dividing the reaction volume into several thousand separated entities allows independent PCR amplification in each single partition, providing a better target to non-target ratio and distributing potential inhibitors across the several thousand reactions (instead of one), resulting in enhanced detection and less inhibition. Subsequently, analytical sensitivity can be enhanced and more accurate quantification achieved without affecting the limit of detection (LoD) for multiplex PCR assays.

PCR reaction partitioning can be generated by different methods, including microdroplets in oil suspension, microwells, or microfluidic valving (13), and ideally, no more than half of the partitions contain a copy of the target sequence to allow optimal quantification via endpoint analysis and absolute determination. Digitally quantifying the total number of positive and negative partitions enables the original concentration of target molecules to be calculated using Poisson statistical analysis (14). As target molecules are divided into different partitions, linked targets, e.g., ribosomal DNA clusters, should be separated by digestion using appropriate restriction enzymes. While in dPCR, microfluidic plates with hundreds or thousands of partitions achieve absolute quantification, ddPCR uses a water-oil emulsion to create thousands of droplets, each acting as a separate PCR reaction chamber.

In this bi-center study, we transferred two well-validated local qPCR assay protocols to detect *Aspergillus* spp. into a digital format, respectively, and tested clinical and contrived samples (artificial samples containing quantified DNA concentrations) to evaluate and compare the new assay formats in both centers. Given that both qPCR assays had been previously optimized and validated both analytically and clinically, it was hoped that the transfer to a digital platform would require little adaptation to the PCR reaction composition and running conditions, although this would likely be platform dependent.

## RESULTS

### Performance when testing contrived serum samples

Of the four different PCR protocols used to test the contrived serum panel (*n* = 30), three PCR protocols generated 100% true positivity rates (TPR) (ddPCR-University Clinic Wuerzburg [UKW], qPCR-UKW, dPCR-Public Health Wales [PHW]). One assay (qPCR-PHW) generated a 96.3% TPR, missing 1/3 samples containing DNA extracted from *Aspergillus flavus* at the lowest fungal burden (one genome/sample). Positive percent agreement (PPA) was 100% between the ddPCR-UKW/qPCR-UKW and the ddPCR-UKW/dPCR-PHW assays and was 96.3% for qPCR-PHW/dPCR-PHW. Across all four PCR protocols, no false positivity was documented, generating 100% analytical specificity and 100% negative percent agreement between all assays.

**Fig 1:**
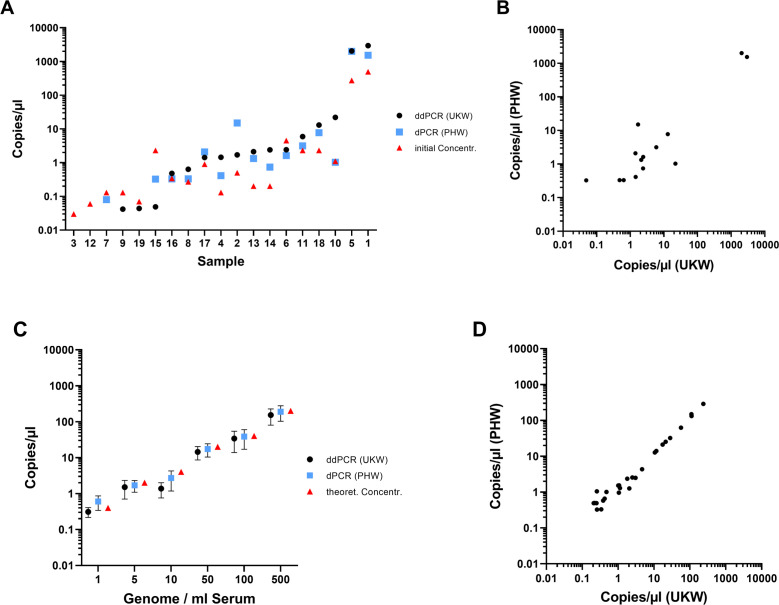
(**A**) The correlation between fungal burden (genomes/mL of sample) in each of the contrived samples and the concentrations determined by the two digital PCR assays when testing the corresponding DNA eluates. When preparing the contrived samples, genome copy numbers per microliter were calculated by converting the measured DNA concentrations in ng/µL (originally determined using the QUBIT3 and HS dsDNA kit) to g/µL and dividing the resulting value by the estimated mass per haploid genome (g). Each species genome mass was estimated using a published reference genome size (bp) (*A. flavus* 36.9 Mbp [NRRL3357, NCBI], *Aspergillus fumigatus* 29.4 Mbp [Af293, NCBI], *Aspergillus niger* 34.9 Mbp [ATCC 1015, JGI]) and multiplying it by the average mass per base pair of double stranded DNA (660 Da) and dividing by Avogadro’s number. The corresponding volume of genomic DNA was spiked into a 500 µL input volume, eluted in 75 µL. The mean values and standard deviations of the detected DNA copy numbers when testing each sample are compared to the DNA concentration used to spike each of the contrived samples. (**B**) A scatter plot presenting the correlation between the DNA concentration (copies/µL) as calculated by both dPCR assays when testing the contrived samples. Each point on the plot represents a paired observation of copy numbers from the two centers when testing the corresponding contrived sample. The correlation between the concentrations determined by both tests was excellent (Spearman’s rho = 0.9605 [95% CI 0.9121 to 0.9825, *n* = 27], *P* < 0.001). (**C**) Comparison of the DNA concentrations as determined by each of the dPCR assays when testing the clinical samples, where the initial DNA concentration was calculated according to the original Cq value, is described in Table 2. Copy numbers of zero (samples 3, 12, 7, 9, 19) are not shown graphically. (**D**) A scatter plot presenting the correlation between the DNA concentration (copies/µL) as calculated by both dPCR assays when testing the clinical samples. Each point on the plot represents a paired observation of copy numbers from the two centers when testing the corresponding contrived sample. The correlation between the concentrations determined by both tests was very strong (Spearman’s rho = 0.887 [95% CI 0.718 to 0.957, *n* = 19], *P* < 0.001).

For the ddPCR/dPCR assays, there was a significant correlation between the concentration calculated by the digital PCR platforms and the fungal burden within the contrived samples (Fig. 1A). The copy numbers of the digital PCR from the two centers were compared with each other and analyzed using Spearman correlation (Table 1; Fig. 1B). The Spearman correlation coefficient (rho) was 0.9605 (95% CI 0.9121 to 0.9825, *n* = 27) with a *P* value <0.001, indicating a statistically significant strong positive correlation.

**TABLE 1:** Detection of contrived sera samples tested at both centers using digital and real-time *Aspergillus* PCR assays^*d*^

Fungal load^*a*^ per sample (ge/sample)	Theoretical DNA concentration per eluate copies/μL^*b*^	Panel composition	PCR assay (Center)									
			ddPCR (UKW)			dPCR (PHW)			qPCR (UKW)		qPCR (PHW)	
			Concentration reported (copies/μL)^*c*^	Number of positive droplets^*c*^	Positivity rate (n/N)	Concentration reported (copies/μL)^*c*^	Number of positive droplets^*c*^	Positivity rate (n/N)	Crossing point (Cq)^*c*^	Positivity rate (n/N)	Crossing point (Cq)^*c*^	Positivity rate (n/N)
0	0.0	(*n* = 3)	0.0 (0.0)	0 (0.0)	0%	0.0 (0.0)	0 (0.0)	0%	Undetermined	0%	Undetermined	0%
1	0.4	**Overall (*n* = 9**) *A. fum.* (*n* = 3) *A. flavus* (*n* = 3) *A. niger* (*n* = 3)	**0.3 (0.1)** 0.4 (0.2) 0.3 (0.1) 0.3 (0.1)	**7.3 (2.2)** 9.0 (2.6) 6.7 (2.1) 6.3 (1.5)	**100%** 3/3 3/3 3/3	**0.6 (0.3)** 0.9 (0.2) 0.5 (0.1) 0.4 (0.1)	**7.3 (3.2)** 11.0 (2.6) 5.7 (1.5) 5.3 (1.2)	**100%** 3/3 3/3 3/3	**40.0 (2.7)** 38.3 (0.4) 41.2 (4.3) 40.4 (1.9)	**100%** 3/3 3/3 3/3	**34.8 (1.0)** 35.1 (0.9) 35.1 (0.0) 34.3 (1.6)	**88.9%** 3/3 2/3 3/3
5	2.0	**Overall (*n* = 6)** *A. fum.* (*n* = 2) *A. flavus* (*n* = 2) *A. niger* (*n* = 2)	**1.5 (0.8)** 2.4 (0.9) 1.1 (0.1) 1.0 (0.0)	**35.3 (19.3)** 57.0 (21.2) 25.0 (1.4) 24.0 (1.4)	**100%** 2/2 2/2 2/2	**1.7 (0.6)** 2.4 (0.1) 1.1 (0.2) 1.5 (0.0)	**20.2 (7.2)** 29.0 (1.4) 13.5 (2.1) 18.0 (0.0)	**100%** 2/2 2/2 2/2	**37.1 (1.4)** 35.8 (0.2) 37.8 (1.3) 37.6 (1.6)	**100%** 2/2 2/2 2/2	**31.9 (0.5)** 31.4 (0.7) 32.2 (0.1) 32.2 (0.4)	**100%** 2/2 2/2 2/2
10	4.0	**Overall (*n* = 3)** *A. fum.* (*n* = 1) *A. flavus* (*n* = 1) *A. niger* (*n* = 1)	**3.1 (1.4)** 4.7(–) 2.1 (–) 2.5 (–)	**72.3 (33.5)** 110 (–) 46 ( –) 61 (–)	**100%** 1/1 1/1 1/1	**2.7 (1.5)** 4.3 (–) 1.3 (–) 2.6 (–)	**32.7 (18.6)** 52 (–) 15 (–) 31 (–)	**100%** 1/1 1/1 1/1	**35.9 (1.5)** 34.4 (–) 37.4 (–) 36.0 (–)	**100%** 1/1 1/1 1/1	**31.2 (0.7)** 30.5 (–) 31.9 (–) 31.2 (–)	**100%** 1/1 1/1 1/1
50	20.0	**Overall (*n* = 3)** *A. fum.* (*n* = 1) *A. flavus* (*n* = 1) *A. niger* (*n* = 1)	**14.4 (5.9)** 21.2 (–) 10.6 (–) 11.6 (–)	**317.3 (116.8)** 452 (–) 243 (–) 257 (–)	**100%** 1/1 1/1 1/1	**17.4 (7.0)** 25.4 (–) 12.7 (–) 14.0 (–)	**208.0 (83.4)** 304 (–) 153 (–) 167 (–)	**100%** 1/1 1/1 1/1	**33.4 (1.0)** 32.3 (–) 34.1 (–) 33.9 (–)	**100%** 1/1 1/1 1/1	**28.8 (0.4)** 28.4 (–) 29.2 (–) 28.8 (–)	**100%** 1/1 1/1 1/1
100	40.0	**Overall (*n* = 3)** *A. fum.* (*n* = 1) *A. flavus* (*n* = 1) *A. niger* (*n* = 1)	**34.0 (20.2)** 56.4 (–) 17.4 (–) 28.2 (–)	**760.7 (438.0)** 1,248 (–) 243 (–) 257 (–)	**100%** 1/1 1/1 1/1	**38.6 (21.4)** 62.5 (–) 21.1 (–) 32.1 (–)	**459.0 (253.5)** 741 (–) 250 (–) 386 (–)	**100%** 1/1 1/1 1/1	**32.3 (1.2)** 30.9 (–) 33.1 (–) 32.9 (–)	**100%** 1/1 1/1 1/1	**27.6 (1.0)** 26.8 (–) 28.8 (–) 27.4 (–)	**100%** 1/1 1/1 1/1
500	200.0	**Overall (*n* = 3)** *A. fum.* (*n* = 1) *A. flavus* (*n* = 1) *A. niger* (*n* = 1)	**153.3 (73.1)** 237.8 (–) 111.3 (–) 110.9 (–)	**3,464.7 (1,619.3)** 5,333 (–) 2,466 (–) 2,595 (–)	**100%** 1/1 1/1 1/1	**189.9 (86.6)** 289.5 (–) 132.1 (–) 148.2 (–)	**2,212.7 (974.9)** 3,332 (–) 1,549 (–) 1,757 (–)	**100%** 1/1 1/1 1/1	**30.1 (1.2)** 28.7 (–) 30.6 (–) 30.9 (–)	**100%** 1/1 1/1 1/1	**25.6 (0.5)** 25.2 (–) 26.1 (–) 25.5 (–)	**100%** 1/1 1/1 1/1

^*a*^ Genome copy numbers per microliter were calculated by converting the measured DNA concentrations in ng/µL to g/µL and dividing the resulting value by the estimated mass per haploid genome (ge). Each species genome mass was estimated using a published reference genome size (bp) (*A. flavus* 36.9 Mbp [NRRL3357, NCBI], *A. fumigatus* 29.4 Mbp [Af293, NCBI], *A. niger* 34.9 Mbp [ATCC 1015, JGI]) and multiplying it by the average mass per base pair of double stranded DNA (660 Da) and dividing by Avogadro’s number. The corresponding volume of genomic DNA was spiked into a 500 µL input volume, eluted in 75 µL.

^*b*^ Assuming 100% extraction efficiency with an elution volume of 75 µL and each genome containing 30 copies of the ribosomal RNA gene cluster (e.g., 50 genomes per sample equals 0.67 genomes per µL eluate, which equates to 20 copies of RNA gene cluster per µL eluate).

^*c*^ Mean ± (standard deviation). Key: qPCR, quantitative/real-time PCR; PHW, Public Health Wales; UKW, University Clinic Wuerzburg; ddPCR, droplet digital PCR; dPCR, digital PCR. “–”, not available.

^*d*^ Bold in the table highlights the overall results.

### Performance when testing clinical samples

*Aspergillus* DNA was reproducibly detected by both dPCR in 14 clinical samples, with two samples (# nine and 19) positive by ddPCR-UKW only and one sample (# seven) positive by dPCR-PHW only, generating a PPA of 82.4%. The qPCR-UKW assay only detected the 14 clinical samples positive by both dPCR assays, generating a PPA between the qPCR-UKW/ddPCR-UKW of 87.5%. In two samples (# three and 12), no *Aspergillus* DNA was detected by any test, other than the original qPCR assay performed in PHW, where a low DNA concentration was determined (Table 2). Samples that were positive across all assays contained higher DNA concentrations (median 1, range 0.13–496) than samples with discordant results (median 0.13, range 0.07–0.13), and overall negative results (median 0.045, range 0.03–0.06). Samples with discordant positivity were only positive in a single partition by digital PCR (Table 2).

**TABLE 2:** Clinical samples tested by both centers using digital and real-time *Aspergillus* PCR

Clinical sample^*a*^	Disease classification	Sample type	Original qPCR result (PHW) Crossing point (Cq)	Dilution factor applied to eluate	Calculated DNA concentration^*b*^ copies/μL	ddPCR (UKW)		dPCR (PHW)		qPCR (UKW) Crossing point (Cq)	qPCR (PHW) Crossing point (Cq)	Positivity across the PCR systems when retesting the DNA extracts
						Copies/μL	Positive droplets	Copies/μL	Positive partitions			
3	Probable IA	Serum	36	None	0.03	0.00	0	0.00	0	Undetermined	Undetermined	0/3 (0%)
12	CPA	BAL fluid	34	1/2	0.06	0.00	0	0.00	0	Undetermined	NT	0/3 (0%)
7	Unclassified	BAL fluid	33	1/2	0.13	0.00	0	0.08	1	Undetermined	NT	1/3 (33%)
9	Probable IA	BAL fluid	33	1/2	0.13	0.04	1	0.00	0	Undetermined	NT	1/3 (33%)
19	Unclassified	BAL fluid	33	1/4	0.07	0.04	1	0.00	0	Undetermined	NT	1/3 (33%)
15	Probable IA	BAL fluid	29	1/2	2.3	0.05	1	0.33	4	38.3	NT	3/3 (100%)
16	Allergic Aspergillosis	BAL fluid	31	1/5	0.341	0.48	10	0.33	4	36.5	32.6	4/4 (100%)
8	*Aspergillus* tracheobronchitis	BAL fluid	32	1/2	0.27	0.64	15	0.33	4	36.3	NT	3/3 (100%)
17	Allergic Aspergillosis	BAL fluid	29	1/10	0.9	1.43	33	2.08	25	36.6	32.3	4/4 (100%)
4	Probable IA	Serum	34	None	0.13	1.44	32	0.41	5	35.3	34.4	4/4 (100%)
2	Probable IA	Serum	32	None	0.5	1.70	38	14.96	181	34.7	33.8	4/4 (100%)
13	Probable IA	BAL fluid	30	1/10	0.2	2.11	45	1.33	16	35.2	NT	3/3 (100%)
14	Probable IA	BAL fluid	30	1/10	0.2	2.40	55	0.74	9	34.7	NT	3/3 (100%)
6	Probable IA	BAL fluid	28	1/2	4.5	2.42	55	1.62	20	35.5	32.9	4/4 (100%)
11	CPA	BAL fluid	29	1/2	2.3	5.91	132	3.14	37	33.4	31.3	4/4 (100%)
18	Probable IA	BAL fluid	28	1/4	2.3	13.04	307	7.74	93	32.4	NT	3/3 (100%)
10	Probable IA	BAL fluid	30	1/2	1.1	22.07	504	1.02	12	31.3	29.7	4/4 (100%)
5	Proven *Aspergillus* sinusitis	Tissue	21	1/5	273	2,042.22	31,826	1,999.38	15,634	24.6	NT	3/3 (100%)
1	Probable IA	BAL fluid	27	1/2	496	2,951.11	39,611	1,534.46	13,258	24.1	22.4	4/4 (100%)

^*a*^ Listed in ascending ddPCR (UKW) concentrations.

^*b*^ DNA concentration was calculated by diluting the initial concentration derived from the original Cq value that was used to calculate a concentration using the linear equation from a robust standard curve (replicates for each concentration *n* = 800, coefficient of variation range 2.0%–4.7%) with standards included in every PHW qPCR run. Key: CPA, chronic pulmonary aspergillosis; IA, invasive aspergillosis; BAL, bronchoalveolar lavage; qPCR, quantitative/real-time PCR; Cq, quantification cycles; PHW, Public Health Wales; UKW, University Clinic Wuerzburg; ddPCR, droplet digital PCR; dPCR, digital PCR; NT, not tested.

The digital PCR results from the two centers typically corresponded with the DNA concentration derived from qPCR (Fig. 1C), and there was a strong positive correlation between the concentrations calculated by both dPCR assays with a Spearman correlation coefficient (rho) of 0.887 (95% CI 0.718 to 0.957, *n* = 19 *P* value < 0.001; Fig. 1D). Fourteen of the 17 samples originating from patients classified with the various manifestations of aspergillosis were positive by the dPCR performed in PHW compared to 15/17 using the UKW assay.

### Performance when testing negative controls

At UKW, two of the sera samples from the 30 healthy controls were positive by ddPCR, with a fungal load of 0.03 and 0.16 copies/μL, generating a true negativity rate of 93.3%. For these false positive samples, one was positive in a single partition in only one of the two replicates, while the other was positive in both replicates, detected in four and one partition, respectively.

Out of the 72 no-template water controls tested at UKW, three were false positive (4.2%), resulting in a true negativity rate of 95.8%. The false positive samples contained one (*n* = 2) and two (*n* = 1) positive droplets, with corresponding calculated fungal loads of 0.06 (*n* = 2) and 0.14 (*n* = 1) copies/µL.

At PHW, seven of the 33 samples from patients with no evidence of fungal disease were positive, generating a true negativity rate of 78.8%. The fungal loads calculated by the QIAcuity software were 0.05 (*n* = 4), 0.06 (*n* = 2), and 0.11 (*n* = 1) copies/µL. All positive samples had a low number of positive partitions, most being positive in a single partition (*n* = 6), with one sample positive in two partitions.

Out of the 28 no-template water controls tested at PHW, five were positive, generating a true negativity rate of 82.1%. All positive samples only had a single positive partition and a calculated concentration of 0.05–0.06 copies/µL.

### The capacity for dPCR to provide species differentiation

**Fig 2:**
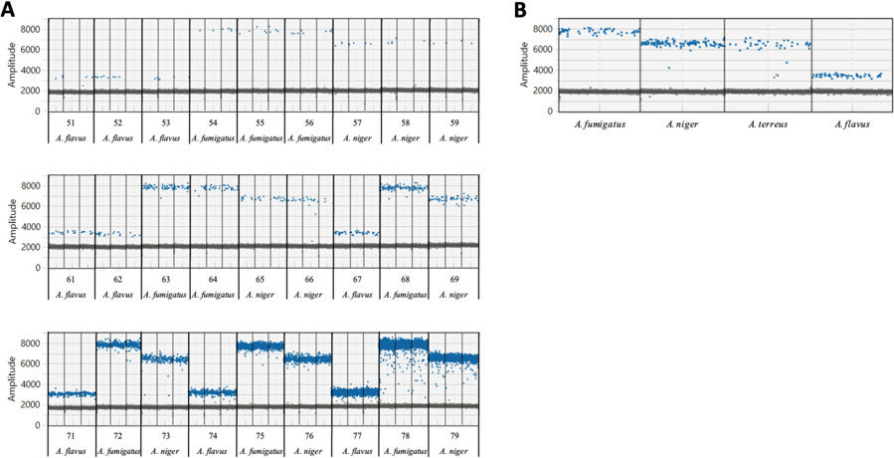
Species differentiation based on the fluorescence amplitude of the positive droplets in UKW ddPCR. (**A**) Dotplot diagram of the contrived samples was compared to (**B**) levels of fluorescence amplitude of reference *Aspergilli*.

ddPCR testing of a selection of reference *Aspergilli* (genomic *Aspergillus* DNA of *A. fumigatus, A. niger, A. terreus,* and *A. flavus*) revealed that the UKW ddPCR assay showed differential levels of the fluorescence amplitude of the different *Aspergilli*. Droplets containing *A. fumigatus* DNA showed the highest fluorescence amplitude compared to *A. flavus*, which had the lowest amplitude. *A. niger* and *A. terreus* could not be distinguished from each other by their fluorescence amplitude. All contrived samples of this study could be correctly assigned to one of the three fungal species by ddPCR based on the level of the fluorescence amplitude of the reference *Aspergilli* (Fig. 2).

**Fig 3:**
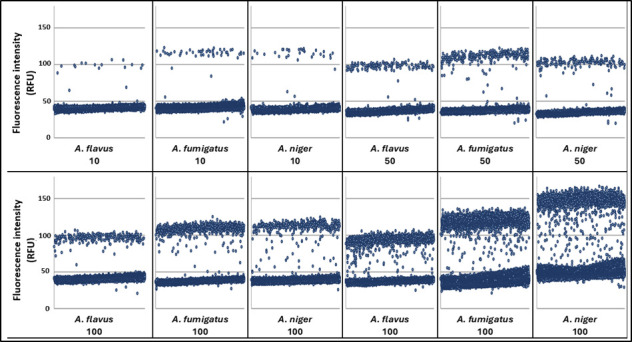
Dotplot diagrams of the fluorescence intensity (RFU) detected by the QIAcuity instrument after testing panel samples of *A. flavus*, *A. fumigatus,* and *A. niger*, with fungal loads of 10, 50, 100, and 500 genomes/0.5 mL sample.

At PHW, across lower burdens (≤100 CFU), it was not possible to differentiate between the species in the panel due to the between-species overlap of fluorescence intensity of the positive partitions (Fig. 3). However, at higher fungal loads (>100 CFU), the different species did appear to be associated with different fluorescent intensities. *A. niger* typically provided the highest fluorescent intensity, followed by *A. fumigatus*, with *A. flavus* associated with the lowest intensity, indicating that the assay may be useful for providing an identification when testing strongly positive BAL fluid or tissue that contained similar fungal burdens (Table 2).

## DISCUSSION

For this bi-center study, we adapted two previously published and well-established qPCR assays to be used as dPCR assays. We assessed the original qPCR protocols from both centers, compared to newly developed dPCR protocols by testing identical contrived and clinical samples, and evaluating performance across all assays.

We revealed an excellent correlation in the fungal burdens between the ddPCR and the dPCR assays (Fig. 1) and an excellent correlation between qPCR burden (based on center-specific Cq values) and the burden determined by both of the dPCR assays, despite utilizing significantly different assays (e.g., oligonucleotides and PCR targets).

Differences between assays extend mainly to samples with very low fungal burden (Table 1), in which individual PCR performances can be quite variable. This includes the analysis of clinical samples, when either dPCR or ddPCR showed positivity only in one partition or droplet, respectively. This observation was independent of the disease classification and was observed in samples from probable IA patients, unclassified patients, and even no-template controls (Table 2). Consequently, the distinction between low-level contamination (false positivity) and true positivity at these low concentrations is difficult and in clinical practice requires the use of additional mycological testing, especially when dPCR and ddPCR generate discordant results (Table 2).

False positivity when testing negative control samples, consisting of ultrapure water or serum samples from healthy persons or patients without any evidence of fungal infection, varied between centers but was typically associated with very low levels of contaminating DNA. At UKW, 2/30 serum samples from healthy individuals were detected as positive (resulting in a true negativity rate [TNR] of 93.3%). In no-template water controls, 3/72 samples were false positive, resulting in a TNR of 95.8%. These false positive samples contained one (*n* = 2) or two (*n* = 1) positive droplets. Similarly, at PHW, 7/33 samples from control patients with no evidence of fungal disease (TNR: 78.8%, mostly one positive partition), and 5/28 no-template water controls were positive (TNR: 82.1%, all with a single positive partition). These results indicate that defining assay-specific thresholds is highly recommended to eliminate any background noise. Although the theoretical limit given by the Poisson statistic is 3 copies per sample (“Rule of the Three” [15]) for a single replicate, detection of <3 partitions can be useful and valid, especially for IFD when testing blood-based samples. Franke et al*.* determined a LoD of 1.96 copies for their ddPCR assay detecting molecular responses in chronic myeloid leukemia patients (16). Applying a positive threshold of >1 positive partition led in our study to a reduced number of positives in unclassified samples (e.g., sample #7 at PHW and sample #19 at UKW) but bears also the risk of potentially false-negative results (UKW: sample #15). Thus, for samples that are in the range of ultra-low positivity, the use of PCR triplicates in dPCR assays and the consideration of a repeat, consecutively collected specimen results are recommended. As with all adjunctive testing, clinical interpretation, along with other mycological evidence, remains paramount.

It has long been known that fungal identification on a species level has an indispensable influence on the selection of the appropriate antifungal drug (17). When using qPCR assays, species differentiation is ensured using specific probes and subsequent melting curve analyses (18). In our study, both dPCR assays allowed the correct identification of different, clinically relevant *Aspergillus* species. While the UKW ddPCR system showed differential levels of the fluorescence amplitude of various *Aspergilli* in different concentrations, the PHW assay reliably detected the different species only at higher concentrations (>100 CFU, Fig. 2 and 3). Such high levels can be found typically in BALF. There is the caveat that many more fungal species might show positivity at identical amplitude levels; in consequence, there is no exclusivity for these fungal species described in this study to be positive at the respective amplitude levels.

The capability of dPCR to quantify exactly even low-abundant DNA targets without any standard curve has led to a large variety of applications in the detection of different pathogens. Gutierrez-Aguirre et al*.* showed that dPCR technology offers various advantages, such as high sensitivity, precision, and reproducibility over common molecular diagnostic platforms, which rely on relative quantification (19). The fact that in dPCR assays, amplicons are distributed into partitions, a process that obeys the Poisson distribution, allows accurate and absolute quantification of the target from the ratio of positive and negative signals against all partitions at the end of the reaction. Furthermore, it has been shown in various clinical materials that dPCR is not as susceptible to target inhibition as qPCR assays (20).

The classical ddPCR system (e.g., the QX200 [BioRad]) uses droplet sample partitioning in nanoliter size, which requires multiple instruments (droplet generator, thermocycler, droplet reader). Each 20 µL sample is partitioned into 20k droplets in a water-oil emulsion. Droplets are transferred into a 96-well PCR plate and end-point PCR is performed in a thermocycler. The PCR plate is loaded onto a droplet reader, where droplets stream single file through the reader, which detects fluorescence. Notably, data quality can be affected by droplet shearing, which is caused by thermal oscillation and by the appearance of so-called rain droplets, which result from damaged droplets or irregular droplet size. dPCR systems, such as the QIAcuity (Qiagen), partition samples into nanowells rather than droplets, and partitioning, thermocycling, and imaging are performed in a single automated instrument. The QIAcuity has a similar workflow to a qPCR experiment; the system has a fully automated partitioning, thermocycling, and imaging, suited to use in routine diagnostic laboratories. The system requires the use of proprietary master mix, nanoplates, and elastic seals but easily accommodates the transfer of qPCR assays. In our study, each 40 µL reaction was divided into up to approx 26k partitions in a 24-well plate. In a direct comparison of dPCR and ddPCR, Tumpach et al*.* demonstrated that the dPCR platform enabled sensitive and accurate quantification similar to the ddPCR system but with greater speed and efficiency (21).

Duplex-ddPCR assays reduce costs and enable quantification of two targets in the same reaction. Beyond that, dPCR assays have the potential to be used as higher-order multiplex assays using multiple primer/probe sets, normally for four targets based on a two-color dPCR detection system (22), and very recently, for even up to 12 targets in an eight-channel system (QIAcuity DXOne [Qiagen]). Quadruplex approaches have been previously described, among others, for the quantitative detection of tuberculosis and resistance-conveying mutations concurrently (23). Referring to this publication, quadruplex dPCR could be used in the future for the parallel detection of *A. fumigatus* and azole-resistance mutations in *CYP51* and other genes.

Our study has certain limitations. This includes a relatively small number of contrived samples and clinical samples from patients with *Aspergillus* infection, and only limited sample exchange between both centers, respectively. Furthermore, some of the negative control samples (sera, water) showed positive results in our *Aspergillus* dPCR assays (TNR varied between 78.8% and 95.8%; however, all these samples had only one positive partition/≤2 positive droplets).

In conclusion, this standardized bi-center study shows that qPCR and dPCR assays yielded comparable results, with dPCR performance being consistent across assays and platforms. As with qPCR, low-level false positivity remains an issue, and the development of positivity thresholds or strategies (triplicate testing/follow-up samples/combination with antigen testing) will be required prior to implementation into routine diagnostic settings. In addition, further comparative validation in large multi-center studies is warranted.

## MATERIALS AND METHODS

### Study design and samples

#### Study setting

The study was performed in the last 6 months of 2023 by two laboratories with extensive experience (>20 years) in performing *Aspergillus* qPCR in a routine diagnostic setting, following established and accredited standardized operating procedures and incorporating the use of both internal and external quality control/assessment. Detailed technical information is provided below. Clinical and contrived DNA eluates were provided by PHW as described below.

#### Sample sources and classification

A panel of contrived and clinical *Aspergillus* DNA samples was generated at PHW Microbiology, Cardiff, for bi-center digital and qPCR testing. The contrived samples consisted of a series of sera (*n* = 30), which were spiked with different concentrations of genomic DNA (gDNA) from three *Aspergillus* species (*A. fumigatus, A. flavus, A. niger)* originally isolated from clinical samples (Table 1). The concentration of the undiluted gDNA source had been determined using a Qubit 3.0 Fluorometer with the Qubit HS dsDNA assay kit following the manufacturer’s instructions. DNA was extracted from 0.5 mL of each of the contrived samples using the BioMerieux eMag generic 3.0.4 protocol with nucleic acid eluted in 75 µL. Each sample eluate within the panel was divided into equal aliquots for anonymous testing at both centers. The panels were stored frozen at −80°C until one panel was sent to UKW on dry ice, while the PHW panel remained in storage until testing.

The clinical samples (*n* = 19) consisted of DNA extracted from clinical samples (BAL [*n* = 15], serum [*n* = 3], tissue [*n* = 1]) that were positive by *Aspergillus* qPCR (Cq range: 21–36 cycles), previously performed as part of routine diagnostic testing. The DNA concentrations (copies/µL) for each positive sample were calculated using a standard curve generated across more than 550 diagnostic runs (coefficient of variation 1.7%–4.1% across the DNA standards). To provide sufficient DNA for qPCR testing at UKW and dPCR at both UKW and PHW, 16/19 extracts were diluted using molecular-grade TE buffer, and the DNA concentration subsequently adjusted. Where sufficient eluate remained, repeat qPCR was also performed at PHW (*n* = 9). The clinical samples originated from 19 patients (M/F ratio: 11/8; age range 5–83 years, median 68 years) with IA (*n* = 11), chronic pulmonary aspergillosis (CPA) (*n* = 2), ABPA (*n* = 2), *Aspergillus* sinusitis, *Aspergillus* tracheobronchitis, with two patients not achieving a final diagnosis of fungal disease. Fungal disease was classified using international consensus definitions or was based on a clinical diagnosis using mycological evidence other than the digital PCR result.

In PHW, all patients (both cases and controls) were initially tested by *Aspergillus* qPCR as part of routine diagnostic testing at the request of the consulting clinician. The extracted nucleic acid was then retrospectively and anonymously tested as a performance evaluation of the *Aspergillus* PCR assays on the digital PCR platforms, with no impact on patient management and not requiring ethical approval.

#### Negative controls

When testing the contrived panel, negative samples (*n* = 3) comprising the same matrix as the positive samples but not spiked with *Aspergillus* genomic DNA served as negative controls subjected to both nucleic acid extraction and PCR amplification. In addition, when performing PCR amplification (both qPCR and dPCR) on the contrived sample eluates, no-template controls (molecular-grade water) were included to monitor each PCR assay. To confirm that positivity was not likely associated with procedural contamination when testing clinical samples, both negative extraction controls and no-template controls were included when initially testing all the samples by qPCR in PHW. As further PCR testing was performed on previously extracted DNA with positivity confirmed as correct, only no-template controls were included in any further PCR testing at PHW and UKW. Additional samples from healthy donors (UKW) or from patients with no evidence of aspergillosis testing negative by qPCR (PHW) were also batch tested by dPCR.

At UKW, 30 sera from two healthy donors (UKW ethics approval #225/12) were subjected to *Aspergillus* DNA extraction using the QIAamp UltraSens Virus Kit (Qiagen) and were tested by ddPCR in duplicates of 9 µL. To avoid airborne contamination, the manual extraction steps were performed in a category 2 laminar flow cabinet. Furthermore, 72 DNA-free water (Carl Roth, Germany) controls were tested by ddPCR.

At PHW, dPCR was performed on clinical samples (*n* = 33) that had tested negative by *Aspergillus* qPCR, derived from either serum or plasma of patients with no evidence of fungal disease according to the EORTC/MSGERC definitions (9). Their DNA had been extracted following the in-house protocol described above for contrived samples. Additionally, 28 DNA-free molecular-grade water controls (QIAcuity Probe PCR kit, Qiagen) were tested on the QIAcuity platform to determine the false positivity rate of the PCR process independent of the influence of the nucleic acid extraction protocol.

### PCR amplification

Two in-house qPCR assays specific for *Aspergillus* spp. and compliant with FPCRI recommendations (24, 25) were adapted to digital PCR using a QX200 system (Bio-Rad) in UKW and a QIAcuity system (Qiagen) in PHW.

#### qPCR assay at UKW

At UKW, qPCR was performed as previously described (25). Briefly, 20 µL reaction mixtures contained 300 nM primer Asp fum_F degen, 300 nM primer Fungi 5.8_R, 150 nM hydrolysis probe ITS-PF, 10 µL of Takyon ROX Probe 2× MasterMix UNG (Eurogentec), and 5 µL of template DNA. Amplification was carried out with a StepOnePlus machine (Applied Biosystems), with the following steps: 50°C for 2 min, 95°C for 3 min, and 60 cycles of 95°C for 5 s, 54°C for 15 s (detection), and 72°C for 1 s.

#### qPCR assay at PHW

At PHW, the *Aspergillus* qPCR test was performed as a single-round assay using a Rotor-Gene Q high-resolution melting instrument (Qiagen, United Kingdom) targeting the 28S rRNA gene, as previously described (24). In each PCR reaction, 15 µL of DNA template was tested in a 25 µL final reaction volume containing 800 nM each primer, 400 nM hydrolysis probe, 4 mM MgCl_2_, 2.5 µL 10xRoche LightCycler hybridization master mix (Roche, United Kingdom), and 2 µL of PCR-grade water. PCR controls in the form of cloned PCR products (300, 30, and 3 input copies) and no-template controls were included in every run to monitor PCR performance. PCR cycling conditions were 1 hold at 95°C for 15 min, followed by 50 cycles at 95°C for 15 s and 60°C for 30 s. An inhibition control and nucleic acid extraction efficiency control assay targeting the quantified *Neisseria meningitidis CTRA* gene was performed independent of the *Aspergillus* qPCR.

#### ddPCR adaptation and ddPCR protocol at UKW

For ddPCR adaptation, different cycling protocols (2- and 3-step) and a gradient of the annealing/elongation temperature were tested to determine the best performance. Takyon ROX Probe 2× MasterMix UNG was replaced with ddSupermix for Probes 2× (Bio-Rad). qPCR probes labeled with 5′ 6-carboxyfluorescein (FAM) and 3′-carboxytetramethylrhodamin (TAMRA) were changed to FAM—Black Hole Quencher 1 (BHQ1 [5′FAM-CAGCGAAATGCGATAAGTAATGTGAATTGCA-BHQ]) to detect *Aspergillus* and hexachloro-*fluorescein* (HEX)—BHQ1 [5′-HEX-AGAGTGACAGGTGGTGCATGGTTGTC-BHQ] to detect *Bacillus,* facilitating duplex PCR reaction. Concentrations of primers and probes in ddPCR settings were increased to 900 and 250 nM, respectively, compared to qPCR (25). The master mix was spiked with 0.1 µL *Bac* template DNA per reaction mixture.

For ddPCR, a 20 µL reaction volume (containing 9 µL of *Aspergillus* template DNA) was digested (Xho I, 10 units) for 30 min at room temperature, mixed with 60 µL oil (Bio-Rad), and up to 20,000 droplets were generated in a QX200 droplet generator (Bio-Rad). Droplets were transferred to a 96-well plate, heat-sealed, and PCR amplified (C1000 Touch Thermal Cycler, Bio-Rad) under the following conditions: 95°C for 10 min, 45 cycles of 95°C for 30 s, 56°C for 60 s and 72°C for 15 s, and 98°C for 10 min (2°C ramping speed) and hold at 10°C. Fluorescence levels were determined by using a QX200 Droplet Reader (Bio-Rad).

Samples were tested in triplicate using 5 and 9 µL of DNA template for qPCR and ddPCR, respectively. A second assay (mold-independent *Bacillus* target [25]) used as a DNA extraction and PCR inhibition control assay in qPCR, but run in separate wells (monoplex), was used in ddPCR in a duplex reaction. Positive PCR controls (genomic *A. fumigatus* DNA) and no template controls were included in each run to monitor PCR performance. The QX Manager Software 2.1 was used for ddPCR result analysis. The ddPCR copy numbers were generated by merging the positive and negative droplets from the replicates and analyzing them combined as a single experiment (26).

#### dPCR adaptation and dPCR protocol at PHW

The dPCR adaptation was carried out in a One-plate QIAcuity 5plex Digital PCR instrument (Qiagen). Reaction mixes were prepared according to the manufacturer’s instructions using the QIAcuity Probe PCR master mix (Qiagen). The primer and probe concentrations were identical to those used in qPCR. Samples were tested in a single round format, adding 26 µL DNA extract per reaction. A final reaction volume of 40 µL was loaded into each well of a 26k 24-well nanoplate (QIAGEN). PCR conditions were 1 hold at 95°C for 2 min, followed by 50 cycles at 95°C for 15 s and 60°C for 30 s. The QIAcuity software suite version 2.2.0.26 was used for result analysis.

### *Aspergillus* species differentiation using ddPCR/dPCR

After spiking of designated samples with three different *Aspergillus* spp. (*A. fumigatus, A. flavus, A. niger*), ddPCR dotplot diagrams were analyzed to determine whether the fluorescence amplitude of the positive droplets in ddPCR allowed differentiation of the three different *Aspergillus* spp. Fluorescence amplitude levels of the four reference *Aspergilli* (genomic *Aspergillus* DNA of *A. fumigatus, A. niger, A. terreus, and A. flavus*) were then used to differentiate the blinded testing of the contrived samples provided by PHW (*n* = 30).

At PHW, the range of fluorescence intensity shown across the positive partition populations according to species was compared to determine the feasibility of species differentiation with QIAcuity dPCR.

### Statistical analyses

PCR positivity was determined according to the reported digital PCR copy numbers and qPCR quantification cycle (Cq value). The TPR was calculated when testing the contrived samples containing *Aspergillus* DNA by dividing the number of positive PCR results by the total number of positive samples. The TNR was determined by applying equation 1 minus the false positivity rate when testing contrived samples with no fungal load, no template controls (i.e., molecular grade water), and human samples where IA was not evident. The correlation between the copy numbers (copies/μL) found at UKW and PHW was analyzed by Spearman correlation using GraphPad Prism (version 9.5.1). Qualitative agreement between positive and negative results generated by the various paired platforms was calculated when applicable.
